# Cortactin and phagocytosis in isolated Sertoli cells

**DOI:** 10.1186/1477-5751-4-11

**Published:** 2005-12-20

**Authors:** Katja M Wolski, Edward Haller, Don F Cameron

**Affiliations:** 1 Department of Anatomy, University of South Florida College of Medicine, 12901 Bruce B. Downs Blvd., MDC6, Tampa, FL 33612, USA; 2 Department of Pathology, University of South Florida College of Medicine, 12901 Bruce B. Downs Blvd., MDC6, Tampa, FL 33612, USA

## Abstract

**Background:**

Cortactin, an actin binding protein, has been associated with Sertoli cell ectoplasmic specializations *in vivo*, based on its immunolocalization around the heads of elongated spermatids, but not previously identified in isolated Sertoli cells. In an *in vitro *model of Sertoli cell-spermatid binding, cortactin was identified around debris and dead germ cells. Based on this observation, we hypothesized that this actin binding protein may be associated with a non-junction-related physiological function, such as phagocytosis. The purpose of this study was to identify the presence and distribution of cortactin in isolated rat Sertoli cells active in phagocytic activity following the addition of 0.8 μm latex beads.

**Results:**

Sertoli cell monocultures were incubated with or without follicle stimulating hormone (FSH; 0.1 μg/ml) in the presence or absence of cytochalasin D (2 μM), as an actin disrupter. Cortactin was identified by standard immunostaining with anti-cortactin, clone 4F11 (Upstate) after incubation times of 15 min, 2 hr, and 24 hr with or without beads. Cells exposed to no hormone and no beads appeared to have a ubiquitous distribution of cortactin throughout the cytoplasm. In the presence of cytochalasin D, cortactin immunostaining was punctate and distributed in a pattern similar to that reported for actin in cells exposed to cytochalasin D. Sertoli cells not exposed to FSH, but activated with beads, did not show cortactin immunostaining around the phagocytized beads at any of the time periods. FSH exposure did not alter the distribution of cortactin within Sertoli cells, even when phagocytic activity was upregulated by the presence of beads.

**Conclusion:**

Results of this study suggest cortactin is not associated with peripheralized actin at junctional or phagocytic sites. Further studies are necessary to clarify the role of cortactin in Sertoli cells.

## Background

The actin binding protein cortactin [1-3] is believed to be involved with actin related cellular events, such as cell motility, cell adhesion, cytokinesis, endo- and phagocytosis, movement of intracellular particles through the cytoplasm, and organization of transmembrane proteins [4]. Clearly Sertoli cells are phagocytic and phagocytize, among other things, residual bodies, apoptotic germ cells, necrotic germ cells, and tubulobulbar complexes [5-9]. Likewise, Sertoli cells isolated from pre-pubertal rat testes have been marked for later detection by the phagocytosis of latex beads [10]. Cortactin crosslinks F-actin *in vivo *[11], is a substrate for the *src *tyrosine kinases [12], and can bind to several other proteins (e.g., ZO-1), thereby possibly stabilizing actin networks [3,13].

Alterations in the cortical cytoskeleton are observed in phagocytosis [1], and actin has been implicated in the endocytic process [14-16]. Cortactin is recruited to the actin-rich membrane ruffles of the entry structure of *Shigella flexneri *when invading HeLa cells and is also found in the periphery of the phagosome shortly after internalization [17]. However, cortactin was not found to be associated with the F-actin in stress fibers [1,17]. Double-labeling experiments in HeLa cells invaded with *S. flexneri *showed a near perfect co-localization of cortactin with actin in the entry structure and at the periphery of the phagosome [17].

Although the presence of cortactin in Sertoli cells has been addressed relative to its role in cell-cell adhesion [18], its role in phagocytosis has not yet been investigated. The current study examined the role of cortactin relative to the cell's phagocytic function *in vitro*.

## Results

All cultures were immunostained for cortactin and not counterstained. Results differential interference contrast microscopy indicated that beads were localized within the cells. There was no attempt to quantify the immunoreaction product, although relative amounts were determined. The presence or absence of FSH had no apparent effect on the distribution or localization of cortactin within Sertoli cells.

### 15 min

**Figure 1:**
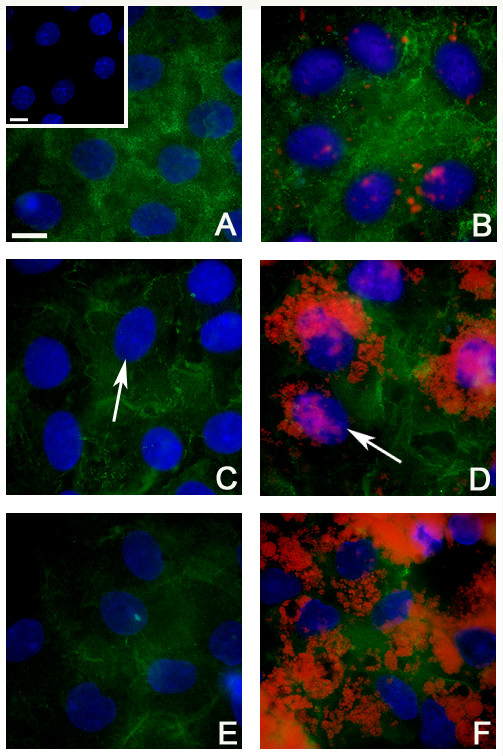
**Fluorescent immunostaining of cortactin in isolated rat Sertoli cells plated on Matrigel^®^**. Sertoli cell monocultures were incubated without (A,C,E) and with (B,D,F) latex beads for 15 min (A,B), 2 hr (C,D), and 24 hr (E,F) and immunostained for cortactin. The negative staining control (A inset) was a primary antibody deletion. Cortactin = green; latex beads = red; Sertoli cell nuclei = blue (arrows also indicate this). Bar = 10 μm.

Cortactin immunostaining was diffuse throughout the Sertoli cell cytoplasm when incubated without beads (Fig. 1A). When incubated with beads, the pattern of intracytoplasmic staining was less diffuse and displayed areas of variable density (Fig. 1B). Cortactin immunostaining was not apparent around the phagocytized beads (Fig. 1B). The primary antibody deletion staining control resulted in the absence of reaction product (Fig. 1A inset).

### 2 hr

Cortactin immunostaining was distributed throughout the cytoplasm of Sertoli cells cultured without beads (Fig. 1C) and with beads (Fig. 1D) and in both was less diffuse than observed in the 15 min cultures. Also, in both treatment groups, reaction product appeared denser at the periphery of the cell (Fig. 1C,D) than observed in the 15 min cultures (Fig. 1A,B). Cortactin immunostaining was not apparent around the phagocytized beads (Fig. 1D).

### 24 hr

**Figure 2:**
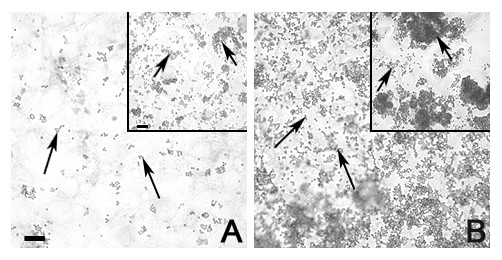
**Peroxidase immunostaining of cortactin in isolated rat Sertoli cells plated on Matrigel^® ^with the addition of 2 μM cytochalasin D and latex beads**. Sertoli cell monocultures were incubated with 2 μM cytochalasin D and latex beads (arrows) for 2 hr (A) and 24 hr (B). At both time periods, the addition of cytochalasin D resulted in an apparent decrease in the amount of beads in Sertoli cell monocultures when compared to Sertoli cell monocultures incubated with beads but without cytochalasin D (inset). These positive controls were incubated for 2 hrs (inset in Fig 2A) and 24 hrs (inset in Fig 2B). Bar = 10 μm.

In cultures without beads, cortactin immunostaining was observed throughout the cytoplasm of the Sertoli cells (Fig. 1E). Although not quantified, there appeared to be less cortactin than that observed at 15 min (Fig. 1A) and 2 hr (Fig. 1C) and less noticeable peripheralization than observed at the 2 hr time period. The addition of beads (Fig. 1F) did not appear to modify the pattern of cortactin immunostaining as compared to the culture without beads (Fig. 1E).

### Cytochalasin D

**Figure 3:**
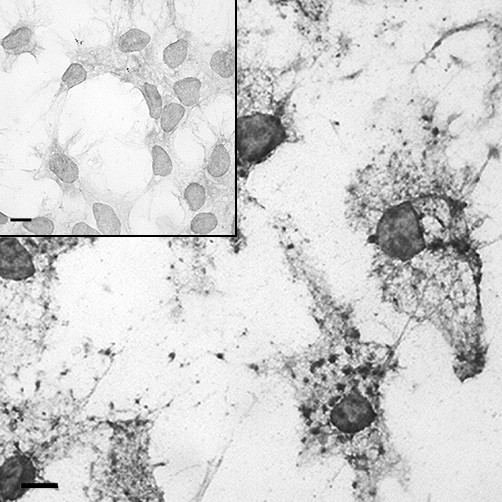
**Peroxidase immunostaining of cortactin in isolated rat Sertoli cells plated on Matrigel^® ^with and without the addition of 2 μM cytochalasin D**. Sertoli cell monocultures incubated with and without (inset) the addition of 2 μM cytochalasin D for 24 hr. The addition of cytochalasin D resulted in a punctate staining pattern of cortactin. Bar = 10 μm.

Some 15 min, 2 hr, and 24 hr cultures were exposed to cytochalasin D. In the 15 min culture, no noticeable difference in the amount of beads was observed. In the 2 hr culture (Fig. 2A), fewer beads were seen in the cultures exposed to cytochalasin D. The amount of beads in the 24 hr culture also appeared to be less than the amount of beads in the cultures not exposed to cytochalasin D (Fig. 2B), as in the 2 hr cultures. In the 24 hr culture, the cortactin reaction product was punctate (Fig. 3), whereas the culture not exposed to Cytochalasin D (Fig. 3 inset) was not, indicating its localization with the cytochalasin D-disrupted F-actin [19].

### Western blot analysis

**Figure 4:**
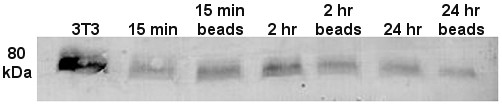
**Immunodetection of cortactin in Sertoli cell lysates from the various time groups probed by anti-cortactin**. Immunodetection of cortactin in Sertoli cell lysates from the various time groups probed by anti-cortactin (p80/p85), clone 4F11 (1 μg/ml; Upstate). The target protein on the membrane was visualized by Western Blue^® ^Stabilized Substrate for Alkaline Phosphatase (Promega).

Western blot analysis (Fig. 4) confirmed the specificity of the primary antibody in control cells (3T3 lysate) and in cultured Sertoli cells.

## Discussion

Cortactin has been associated with endo- and phagocytosis *in vivo *and *in vitro *[4,14-16]. A study by Chapin et al [18] indicated the presence of cortactin near the lumen of seminiferous tubules in stages VII and VIII of the rat seminiferous epithelium cycle, which correlates with the sharp increase in phagocytic activity by Sertoli cells [20,21]. On the basis of this correlation, the current project was designed to determine if cortactin is involved in the phagocytic process of the Sertoli cell, as indicated in HeLa cells [17], in which positive cortactin immunostaining was observed in association with actin in the phagocytosis of *S. flexneri *by these cells.

Results of the current study show that cortactin is found throughout the cytoplasm in isolated Sertoli cells maintained on Matrigel^® ^for up to 96 hours of incubation. Treatment with cytochalasin D confirmed that the cortactin in isolated cells was likely associated with F-actin, since Sertoli cells treated with cytochalasin D showed the same punctate immunostaining of cortactin as observed for actin in cells exposed to this toxin [19]. Likewise, it is well known that cortactin associates with actin in other cells, as reported by Wu and Parsons [1], Urono et al [2], and Weaver et al [3].

The observations of Dehio et al [17] that cortactin appears to be involved in early phagocytosis in HeLa cells suggested that this actin binding protein may also be involved in phagocytosis in Sertoli cells. As observed by Filippini et al [22], isolated Sertoli cells begin phagocytizing beads after 15 min incubation, as observed in the current study, and the rate of phagocytosis plateaus after 5 hr. If cortactin is associated with phagocytic invagination of the cell membrane, early phagosomal formation, and/or phagosomal transport, this protein would likely be localized around beads after 15 min, with an increase in immunostaining at 2 hr. This, however, was not observed in the current study. The lack of apparent correlation between cortactin localization and bead uptake, suggests cortactin is not involved in initial phagocytosis in isolated Sertoli cells.

FSH treatment did not appear to alter the distribution of cortactin within Sertoli cells at any time point observed throughout the treatment period. FSH has been shown to increase the binding of residual bodies and cytoplasts from elongated spermatids to Sertoli cells [6], however, Filippini et al [22] demonstrated that FSH inhibits the actual phagocytic activity of the cells. Our results would suggest that the role of FSH in Sertoli cell phagocytosis is not related to cortactin.

## Conclusion

The distribution of cortactin within Sertoli cells did not appear to be related to FSH at any time point observed, therefore suggesting that the role of FSH in Sertoli cell phagocytosis is not related to cortactin. Cortactin also does not appear to be related to junctional F-actin, in that the peripheralized actin in the Sertoli cell cultures did not appear to include cortactin. Results from this study indicate a need for additional studies to clarify the role of cortactin in the Sertoli cell.

## Methods

### Sertoli cell Isolation, culture, and pretreatment

Sertoli cells were isolated from 16-day-old Sprague-Dawley rats (Harlan) by sequential enzymatic digestion with trypsin and collagenase, as previously described by Cameron et al [23]. Briefly, testes were excised from prepubertal male rats, and the parenchyma was digested with routine sequential enzyme treatments (0.25% trypsin, followed by 0.20% collagenase). Cells were plated (<1.5 × 10^6 ^cells/cm^2^) in 4 well chamber slides (Lab-Tek^®^) precoated with Matrigel^® ^(1:5 dilution with supplemented medium). Sertoli cell viability at plating was >95%. Plated cells were incubated in DMEM:F12 medium supplemented with 50 ng/ml retinol (Acros) and 0.01 cc/ml insulin-transferrin-selenium (ITS; Sigma) at 39°C, in a humidified incubator with 5% CO_2_-95% air, for 2 days, to expedite the removal of contaminating germ cells. The Sertoli cell cultures were then treated with a hypotonic solution of sterile 20 mM Tris-HCl buffer for 2.5 min at 37°C to remove any remaining germ cells, after which the pretreated cells were then placed in a humidified chamber at 33°C with 5% CO_2_-95% air.

### Treatment groups

Pretreated Sertoli cell monocultures were incubated for 24 hours, after treatment with Tris-HCl, in supplemented DMEM:F12 medium and incubated with or without 0.1 μg/ml FSH (NIDDK-oFSH-19-SIAFP, 94× NIH-FSH-S1/mg; gift from NIDDK-NIH) in a humidified chamber at 33°C with 5% CO_2_-95% air for an additional 24 hours.

At time 0 (4 days in culture), some pretreated Sertoli cell monocultures received 2 μM cytochalasin D (Sigma) [24], 0.8 μm latex beads (Sigma), or both 2 μM cytochalasin D and 0.8 μm latex beads. These pretreated Sertoli cell monocultures were incubated for 15 min, 2 hr, or 24 hr in supplemented DMEM:F12 medium in a humidified chamber at 33°C with 5% CO_2_-95% air. No attempt was made to quantify bead uptake by Sertoli cells, although all cultures were observed by differential interference contrast microscopy after fixation and repeated washings to determine if the beads were in or on the cells. Control pretreated Sertoli cell monocultures received no cytochalasin D or latex beads.

### Cortactin immunostaining

Sertoli cells were fixed with 100% ethanol and immunostained for cortactin, as described by Wine and Chapin [25], using 10 μg/ml anti-cortactin (p80/p85), clone 4F11 (Upstate) as the primary antibody. Two secondary antibodies were used. The first one was rat antimouse IgG1 heavy chain:biotin (1:200; Serotec), which was conjugated to streptavidin horseradish peroxidase (Zymed). Positive immunostaining was visualized with bright light by reduced DAB (Vector Labs). The second one was rat antimouse IgG1 heavy chain:FITC (1:200; Serotec). Positive immunostaining was visualized by ultraviolet light. Latex beads fluoresced when excited with 540 nm wavelength light, there was no counterstaining, and appropriate positive (3T3 cells) and negative (primary antibody deletion) staining controls were used.

### Western blot analysis

SDS-PAGE gel electrophoresis was performed to verify the antibody specificity. 20 μg protein was loaded onto the gel. Cold cell lysis buffer (50 mM Tris-HCl, pH 7.4; 1% NP-40; 0.25% sodium deoxycholate; 150 mM NaCl; 1 mM EDTA; Complete™ Mini protease inhibitor cocktail (Roche); 1 mM Na_3_VO_4_; 1 MM NaF) was added to the cell cultures, and the cells were detached using a disposable cell scraper. The cell suspension was lysed on an orbital shaker for 15 min at 4°C, after which the lysate was centrifuged at 14,000 × g for 15 min at 4°C. The supernatant was collected for Western blot analysis.

Lysates were separated on a 7.5% Tris-HCl Ready Gel (BioRad) and transferred to nitrocellulose using a semi-dry blotting apparatus. The membrane was immunostained with 1 μg/ml anti-cortactin (p80/p85), clone 4F11 (Upstate), as the primary antibody, and anti-mouse IgG (H&L) AP conjugate (Promega), as the secondary antibody. The target protein on the membrane was visualized by Western Blue^® ^Stabilized Substrate for Alkaline Phosphatase (Promega).

## Authors' contributions

KMW carried out all experiments. EH aided KMW in carrying out the immunocytochemistry. DFC participated in the design of the study and read and approved the final manuscript.
